# Publications Are More Than Lines on Your Curriculum Vitae: An Epistle to Residents (and Faculty)

**DOI:** 10.7759/cureus.77882

**Published:** 2025-01-23

**Authors:** William C Baumgartner, Michael J Asken, William K Childers

**Affiliations:** 1 General Surgery, UPMC (University of Pittsburgh Medical Center) Harrisburg, Harrisburg, USA; 2 Internal Medicine, UPMC (University of Pittsburgh Medical Center) Harrisburg, Harrisburg, USA; 3 General Surgery/Abdominal Wall Reconstruction, UPMC (University of Pittsburgh Medical Center) Harrisburg, Harrisburg, USA

**Keywords:** clinical communication, critical thinking, medical writing, mentorship in medicine, professional development

## Abstract

Medical professionals often view writing and publishing as a mere checkbox on a curriculum vitae (CV), yet these activities hold profound value beyond career advancement. This article explores the transformative impact of writing on professional and personal development within the medical field, emphasizing how writing refines critical thinking, fosters creativity, and enhances communication skills. Through the example of Dr. Ignaz Semmelweis and parallels from other fields, the article illustrates how writing and publication can influence peers and shape future practices, even without immediate recognition. The article also highlights the evolving role of technology, including conversational AI, in enhancing academic writing and manuscript quality. It underscores the role of writing in patient care, as clear clinical documentation improves patient outcomes by reducing errors and enhancing interdisciplinary communication. Additionally, writing serves as a pathway for discovering hidden talents and interests, as evidenced by the unique career pivot of football coach Andy Reid. This work also highlights the importance of mentorship in guiding novice authors through the rigors of publication and the resilience built through critical feedback. Ultimately, writing contributes far more than CV lines, serving as a powerful catalyst for growth, mastery, and the propagation of innovative ideas within the medical profession.

## Editorial

Benjamin Franklin once wrote, "Either write things worth reading or do things worth writing," a timeless reminder of the power of the written word [1]. In the academic realm of medicine, there is undeniable personal satisfaction and professional recognition in receiving an acceptance email for your paper from a respected journal. However, while acceptance feels like validation, and despite the growing number of publishing options, many still find writing and publishing to be daunting tasks, viewing them as little more than entries on their curriculum vitae (CV). Writing holds far greater significance than simply fulfilling the "publish or perish" mandate and can be a transformative endeavor that enriches both the writer and their profession.

Why write?

Writing is about sharing your expertise, views, and research on topics you value. Whether or not your work is accepted by a prestigious journal, it can make a lasting impact in ways you may never fully realize. Consider the case of Dr. Ignaz Semmelweis, a Hungarian physician whose early publication on the importance of handwashing was largely dismissed by the medical community of his time. Despite this rejection, his work inspired others outside of the traditional medical establishment, who, recognizing the importance of his findings, built upon them. His ideas on antisepsis eventually laid the foundation for modern procedures, saving countless lives. Semmelweis' story demonstrates how writing can influence thinkers and the future, even when not immediately embraced by one's peers or even obvious to you as an author [2].

The primary purpose of writing articles is to share new knowledge, expand the boundaries of the field, and encourage ongoing progress. These insights and reflections are the butterfly wings that propel the tsunami of transformative ideas and revolutionary innovation.

The process of writing, even when it doesn't result in anticipated publication, sharpens cognitive abilities by forcing you to organize your thoughts logically and present them with clarity and conciseness. These skills extend well beyond academic writing and have tangible effects in clinical practice.

For example, well-written clinical notes are vital for enhancing communication between healthcare providers and ensuring continuity of care. Clear and comprehensive notes help reduce errors, improve medication management, and support interdisciplinary collaboration, all of which contribute to improved patient safety and outcomes [3]. Studies also show that clear discharge summaries help reduce readmission rates by ensuring that patients and their caregivers fully understand their treatment plans and follow-up care [4]. By fostering critical thinking and promoting clarity, the skills developed through writing can lead to more thoughtful decision-making, ultimately enhancing the quality of patient care [5].

Engaging deeply in the writing process can lead to experiencing “psychological flow,” the mental state of full immersion in a task that heightens creativity and productivity. When a writer reaches this state, writing becomes deeply satisfying and rewarding [6]. Flow not only enhances the quality of the manuscript, but also provides a sense of accomplishment, contributing to both professional growth and personal well-being.

While writing requires discipline, it also offers a break from formulaic clinical communication routines, such as patient notes and case presentations. It provides a space where creativity can flourish, serving as both a respite and a catalyst for innovative ideas and techniques that can reinvigorate professional practice. A qualitative meta-synthesis highlighted that health professionals and students who engage in reflective writing experience enhanced learning from their experiences, leading to improved patient care and personal development [7].

Recent advancements in conversational AI, such as ChatGPT, are further enhancing the way manuscripts are developed and refined. These tools assist in generating coherent drafts, improving clarity, and streamlining the editing process, making scholarly writing more attainable. Singh et al. highlight how these technologies not only expedite manuscript preparation, but also foster creativity and precision, broadening the scope of academic contributions [8].

In addition to technological advancements, professional medical writers play an indispensable role in the publication process. As highlighted by Khan, these experts bridge the gap between complex medical research and clear, impactful communication [9]. By ensuring accuracy, adherence to journal guidelines, and the ethical presentation of data, professional medical writers enhance the quality and integrity of published works. Their contributions are especially vital in collaborative projects, where they help unify diverse perspectives into cohesive narratives, ultimately advancing the dissemination of medical knowledge [3].

Receiving feedback from reviewers, though sometimes difficult, is a crucial part of the writing process. The resulting professional and personal development strengthens not only the quality of your writing, but also your resilience. The emotional journey through critical feedback or rejection, which might be likened to the stages of grief, ultimately fosters a more refined and impactful manuscript. The process of reflection, as noted in a study on physicians' experiences, is essential for continuous performance improvement and is integral to professional practice [10].

Article development will challenge your knowledge base. While you need not be the expert initially, the process demands a commitment to growth. Mastery and expertise are not innate, they are cultivated through continuous learning and ongoing effort. If your grasp of the topic feels insufficient, it should serve as motivation to acquire knowledge needed to write with confidence and authority.

Moreover, writing promotes continuous learning. The process of researching and synthesizing information, regardless of publication outcome, equips you with the latest evidence and innovations, which you can then apply to your practice. This ongoing engagement with literature ensures that physicians remain at the forefront of their field, ultimately enhancing patient care and outcomes.

Writing can reveal personal strengths and interests that may not emerge in the day-to-day demands of clinical care. Through the process of writing, you may discover new perspectives and capabilities that can reshape your professional path.

A compelling example of this phenomenon comes from outside the medical field. Kansas City Chiefs elite football coach Andy Reid began his career as a college sports journalist. What Reid did not realize at the time was that his perspective on football, shaped by writing, was actually that of a coach. The act of writing about sports uncovered a latent talent for coaching, which ultimately led him to become one of the most successful figures in professional football [11].

Similarly, physicians who engage in writing may uncover hidden talents in research, education, or leadership areas that may not be evident in their daily routine. Authorship provides an opportunity for reflection and exploration, where ideas can be distilled and new interests discovered. In some cases, writing can lead to unexpected career shifts or the realization of passions that lie beyond the clinical setting.

Although some may view mentorship as a separate topic, its importance in developing writing skills cannot be overstated. Effective mentorship provides not only technical guidance on writing, but also provides support and enhances confidence and resilience, crucial elements in facing rejection and refining your work. A good mentor helps articulate your thoughts clearly, improve your writing, and navigate the challenges that come with the publication process. With mentorship, novice writers can learn to persevere in the face of feedback, turning critiques into opportunities for growth and development.

Finally, there is the physiological impact of a well-written, or rather, a poorly written article on your colleagues and audience. While, likely, a little known altruistic impact, having produced a skilled and grammatically correct piece, you will spare others the cardiac stress that has been shown to occur in grammatically erudite individuals when confronting poor grammar [12].

In summary, writing and publishing contribute more than just lines on your CV. They are powerful experiences for professional and personal growth, serving as catalysts for innovation, collaboration, and the dissemination of new ideas. They help sharpen critical thinking, encourage creativity, and, ultimately, enhance the quality of your professional communications and patient care (skills that may atrophy as AI appropriates those functions).

Writing is an opportunity to refine your skills and build your confidence. Writing well matters, not just for publication, but for the broader impact it can have on your skills, career, and the field at large. As Ernest Hemingway aptly stated, "We are all apprentices in a craft where no one ever becomes a master" [13]. This reminds us that writing is a continual journey of growth and discovery, with each piece offering the chance to contribute meaningfully to the world of medicine.
